# A Comparative Study of Morphine and Clonidine as an Adjunct to Ropivacaine in Paravertebral Block for Modified Radical Mastectomy

**DOI:** 10.7759/cureus.42950

**Published:** 2023-08-04

**Authors:** Mukesh Kumar, Rajni Gupta, Prateek Kumar Dinkar, Haider Abbas

**Affiliations:** 1 Emergency Medicine, King George's Medical University, Lucknow, IND; 2 Anesthesiology, King George's Medical University, Lucknow, IND

**Keywords:** ropivacaine, clonidine, morphine, paravertebral block, post-operative pain

## Abstract

Background

General anesthesia (GA) is a standard for breast malignant surgery. The issue of postoperative pain as well as the high occurrence of nausea and vomiting have prompted the quest for a superior methodology for tormenting the executives with fewer complications. Over the most recent couple of years, paravertebral block (PVB) has acquired huge fame either in combination with GA or alone for anesthetic management. In this study, we aim to evaluate the efficacy of morphine and clonidine as an adjunct to ropivacaine in PVB in breast cancer patients undergoing modified radical mastectomy.

Methods

In this study, a total of 90 patients were divided into the following three groups (30 each) based on a computer-generated random table. Group C (control): PVB with 0.25% ropivacaine (19 ml) 1 ml saline; Group M: PVB with 0.25% ropivacaine (19 ml) + 20 microgram/kg body weight morphine; Group N: PVB with 0.25% ropivacaine (19 ml) + 1.0 microgram/kg body weight clonidine. The postoperative pain intensity was recorded using the visual analog scale (VAS), and sedation was observed by the Ramsay Sedation Scale (RSS) score.

Results

The VAS was similar at zero hours, two hours, and four hours in the postoperative period among all the groups. There was a significant (p = 0.003) difference in VAS from six hours to 20 hours in the postoperative period among the groups. A significant (p < 0.05) difference was observed among the groups at eight hours to 20 hours. The first requirement of analgesia was significantly (p = 0.001) higher in Group N (7.70 ± 1.74) than in Group C (4.43 ± 1.43) and Group M (7.33 ± 2.21).

Conclusion

Morphine in the PVB provides better postoperative analgesia. The consumption of rescue analgesia was significantly reduced in the morphine group as compared to the clonidine group. The procedure also proved to be safe as no complication was encountered in the PVB in our study.

## Introduction

Pain is the most dreaded side effect of surgery for the patient, both during and after surgery. It has various physiological side effects, such as increased myocardial oxygen demand for poor ventilator function, high sympathetic tone, decreased urine output, and paralytic ileus, as well as psychological disturbances like anxiety, sleep disturbances, altered behavior, and psychosis. Poorly controlled acute pain can lead to chronic pain syndrome, which is very distressing to the patient. Therefore, control of pain is an important element in the perioperative period and requires exhausting effort from the attending anesthesiologist.

Many treatment modalities have been developed to combat pain and its consequences. These modalities include intravenous (IV) analgesics (opioids and non-opioids), central neuraxial blockade, nerve plexus block, isolated nerve block, and local infiltration. Some non-pharmacological methods like toxic epidermal necrolysis (TENS), acupuncture, and acupressure have been tried with variable success [1].

Hugo Sellheim of Leipzig invented the paravertebral block (PVB) concept in 1905. It was further refined by Lawen (1971) and Kappis (1919) when a renewed interest developed in the topic due to efforts from Eason and Wyatt, who presented a reappraisal of the thoracic paravertebral block (TPVB) [2,3]. TPVB provides high-quality analgesia and is a great advantage for patients undergoing many different surgeries. At the same time, it relieves acute postoperative pain and may prevent the development of chronic pain [4,5]. Traditional pain management has been reported to cause inadequate pain control [6]. The possible complications can be inadvertent vascular puncture, pleural puncture, pneumothorax, epidural or intrathecal spread, failure of the technique, and hypotension. To reduce the complications, many modalities have been introduced, for example, ultrasound-guided techniques, nerve stimulator techniques, and fluoroscopic guidance [7]. Adjuvants like epinephrine, clonidine, fentanyl, morphine, and dexmedetomidine have been used with local anesthetics like ropivacaine, bupivacaine, and lignocaine to improve and extend pain relief after surgery [7-10].

Morphine is the principal alkaloid of opium. Morphine acts as a µ agonist, binding to receptors in the brain, spinal cord, and other tissues. Local anesthetic drugs with adjuvants like fentanyl, morphine, and clonidine have been studied, and they improve the quality of the blockade [11]. Ropivacaine is a local anesthetic that blocks the generation and conduction of nerve impulses, presumably by increasing the postjunctional in the medulla (vasomotor center) threshold for electrical excitation in the nerve, slowing the propagation of the nerve impulse, and reducing the rate of rise of the action potential [12]. In general, the progression of anesthesia is related to the diameter, myelination, and conduction velocity of affected nerve fibers.

Specifically, the drug binds to the intracellular portion of sodium channels and blocks sodium influx into nerve cells, which prevents depolarization [13]. Imidazoline derivatives are partial agonists with high affinity and high intrinsic activity at alpha-2 receptors (alpha-2A subtype) in the brainstem. Major hemodynamic effects result from stimulation of alpha-2A receptors present mainly postjunctional in the medulla (vasomotor center) [14]. In this study, we aim to evaluate the efficacy and usefulness of morphine and clonidine as adjuncts to ropivacaine in PVB in breast carcinoma (CA) patients undergoing modified radical mastectomy.

This article was presented as a meeting abstract at the International Conference on Anesthesiology and Critical Care Medicine (ICACCM 2023), Paris, France, January 23-24, 2023.

## Materials and methods

Study population and design

This case-control, prospective, randomized, single-blind study was conducted at the Department of Anesthesia, King George's Medical University, Lucknow, Uttar Pradesh, India.

Study duration

This study was conducted between 2013 and 2016.

Inclusion criteria

Diagnosed cases of carcinoma breast (CA breast) with American Society of Anesthesiologists (ASA) I and II physical status, aged between 18 and 60 years, and scheduled for elective modified radical mastectomy were included in the study.

Exclusion criteria

Patients with contraindications of the PVB, heart block, psychiatric illness, bleeding disorder, allergy to amide-type local anesthetics, infection at the thoracic paravertebral injection site, body mass index > 35 kg/m^2^, previous ipsilateral thoracic surgery, total pleurectomy, localized tumor, empyema, and abnormal thoracic anatomy were excluded from the study.

Sample size

We enrolled a total of 90 patients who were admitted to our department between 2013 and 2016 based on the well-defined inclusion and exclusion criteria.

Study procedures

After getting approval from the Institutional Ethics Committee (IEC), this case-control, prospective, randomized, single-blind study was commenced. Written informed consent was obtained from each patient. All 90 patients were divided into the following three groups (30 each) on the basis of a computer-generated random table. Group C (control): PVB with 0.25% ropivacaine (19 ml) + 1 ml saline; Group M: PVB with 0.25% ropivacaine (19 ml) + 20 microgram/kg body weight morphine; Group N: PVB with 0.25% ropivacaine (19 ml) + 1.0 microgram/kg body weight clonidine. All patients were given premedication on the night before surgery with ranitidine 150 mg and alprazolam 0.25 mg orally. All of them were properly informed regarding the procedure of giving PVB and were preloaded with 10-15 ml/kg of Ringer lactate (RL). After the patients had been given PVB, the following clinical parameters were monitored: intraoperative and postoperative heart rate, mean arterial pressure (MAP), oxygen saturation (SpO_2_), and complications.

In the operating room (OR), IV access was secured and IV fluid was started. Standard monitoring with a 12-lead electrocardiogram (ECG), non-invasive oscillometric blood pressure (NIBP), and pulse oximeter (SpO_2_) was initiated. All patients were positioned in a sitting position, and the C7 cervical spine was identified and marked, followed by the T4 to T7 vertebrae, respectively. Under aseptic precautions, at 2.5 cm lateral to the cephalad edge of the T4 spinous process, the skin, subcutaneous tissue, and the periosteum of the transverse process of the T4 vertebra were infiltrated with 3 ml of lignocaine 2%. A 25G 10 cm insulated needle was introduced at 90 degrees to the skin, at the site of local anesthetic infiltration. The needle was advanced till it touches the transverse process of the vertebra, noting the depth. The needle was withdrawn and then advanced slightly caudal to walk off the transverse process for a distance of 1.0 to 1.5 cm. The study drug (20 ml), as per the group allocation, was injected in small aliquots of 5 ml with repeated aspiration in between. Any complication or difficulty during the performance of PVB was noted.

Thereafter, general anesthesia (GA) was premedicated with IV ondansetron 4 mg and IV glycopyrrolate 0.2 mg, and then the patients were induced with IV fentanyl 2 µg/kg and IV propofol 2 mg/kg. The orotracheal intubation was facilitated by IV succinylcholine 2 mg/kg and ventilation was controlled. Anesthesia was maintained with IV vecuronium, oxygen and nitrous oxide, and inhalational agents. MAP was maintained within 20% of the preoperative baseline. IV ondansetron 4 mg was administered once the patient was induced. No other analgesics were administered intra-operatively. IV mephentermine 6 mg was administered as needed to keep MAP more than 60 mmHg, and bradycardia was managed by injection of atropine 0.02 mg/kg body weight. At the end of the surgery, residual neuromuscular blockade was reversed with IV neostigmine 50 µg/kg + 10 µg/kg IV glycopyrrolate. Patients were extubated on spontaneous respiration and returned to consciousness. In the postoperative period, we assessed the duration of analgesia, visual analog scale (VAS), Ramsay Sedation Scale (RSS) score, time of first requirement of rescue analgesia, total consumption of rescue analgesia, and any complication in the postoperative period.

In the postoperative anesthesia care unit (PACU), the patients were monitored for two hours. Analgesia, level of sedation, and postoperative nausea and vomiting (PONV) were assessed on arrival to PACU, at zero hours, and at two-hour intervals up to 24 hours, with VAS score, where 0 = no pain and 10 = worst imaginable pain. If the patient complains of pain and VAS is > 3, IV paracetamol 100 ml infusion was administered, and VAS ≤ 3 was maintained during the PACU stay. The patients were given IV paracetamol 100 ml infusions of rescue analgesia in the ward from the time they complain of pain (VAS > 3). The duration of analgesia in minutes counted from the time of initiation of the PVB to the first analgesic request (VAS > 3) was noted.

Nausea was defined as a subjectively unpleasant sensation associated with awareness of the urge to vomit. An event of vomiting was defined as vomiting (forceful expulsion of gastric contents from the mouth) or retching labored, spasmodic, rhythmic contractions of the respiratory muscles without expulsion of gastric contents. PONV was assessed on a three-point numerical rating scale, where 0 = no nausea, no vomiting, 1 = nausea present, no vomiting, and 2 = vomiting present with or without nausea. IV ondansetron 4 mg was administered as a rescue antiemetic if the PONV score was 2 or more.

Statistical analysis

Continuous data were summarized as mean ± SD while discrete (categorical) data in percentage (%). The outcome measures (pulse rate, systolic blood pressure, diastolic blood pressure, SpO_2_, sedation score, and VAS score) of three groups over the periods (time) were compared by repeated measures of two factors (groups x periods) analysis of variance (ANOVA) using general linear models (GLM) followed by Tukey’s post hoc test after ascertaining the normality by Shapiro-Wilk test and the homogeneity of variance by Levene’s test. Groups were also compared by one-way ANOVA followed by Tukey’s post hoc test. The categorical variables were compared by the chi-square (χ^2^) test. A two-sided (α = 2) p < 0.05 was considered statistically significant. All analyses were performed on Statistica version 6 (StatSoft GmbH, Hamburg, Germany).

## Results

The baseline characteristics of the patients are shown in Table 1. The mean age (years), height (cm), weight (kg), and BMI (kg/m^2^) were 44.20 ± 9.37, 157.17 ± 7.14, 62.53 ± 6.17, and 25.43 ± 3.15, respectively, in Group C; 49.17 ± 7.87, 153.3 ± 5.88, 608 ± 5.3, and 25.62 ± 2.87, respectively, in Group M; and 47.50 ± 8.95, 156.83 ± 8.1, 61.3 ± 4.58, and 25.12 ± 3.38, respectively, in Group N. The mean age (years), height (cm), weight (kg), and BMI (kg/m^2^) and distribution of patients according to ASA were not significantly different in between groups.

**Table 1:** Baseline characteristics of the patients (N = 90) ^1 ^ANOVA test; ASA: American Society of Anesthesiologists; BMI: body mass index.

	Group C (N = 30)	Group M (N = 30)	Group N (N = 30)	p-value^1^
	Mean ± SD	Mean ± SD	Mean ± SD	
Age (years)	44.20 ± 9.37	49.17 ± 7.87	47.50 ± 8.95	0.15
Height (cm)	157.17 ± 7.14	153.3 ± 5.88	156.83 ± 8.1	0.15
Weight (kg)	62.53 ± 6.17	60 ± 5.38	61.3 ± 4.58	0.21
BMI (kg/m^2^)	25.43 ± 3.15	25.62 ± 2.87	25.12 ± 3.38	0.91
ASA	N (%)	N (%)	N (%)	
I	16 (53.3)	17 (56.7)	13 (43.3)	0.23
II	14 (46.7)	13 (43.3)	17 (56.7)	

**Figure 1:**
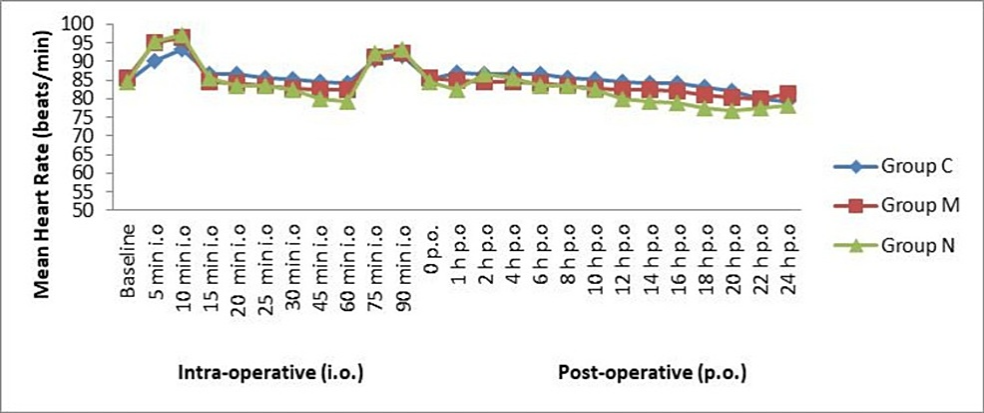
Mean heart rate in intraoperative and postoperative periods (N = 90)

The heart rate was similar at 0 minutes among all the groups (p > 0.05). The heart rate was almost stable in the intraoperative period and postoperative period. No significant (p > 0.05) difference was observed among the groups in heart rate at different time intervals (Figure 1).

**Figure 2:**
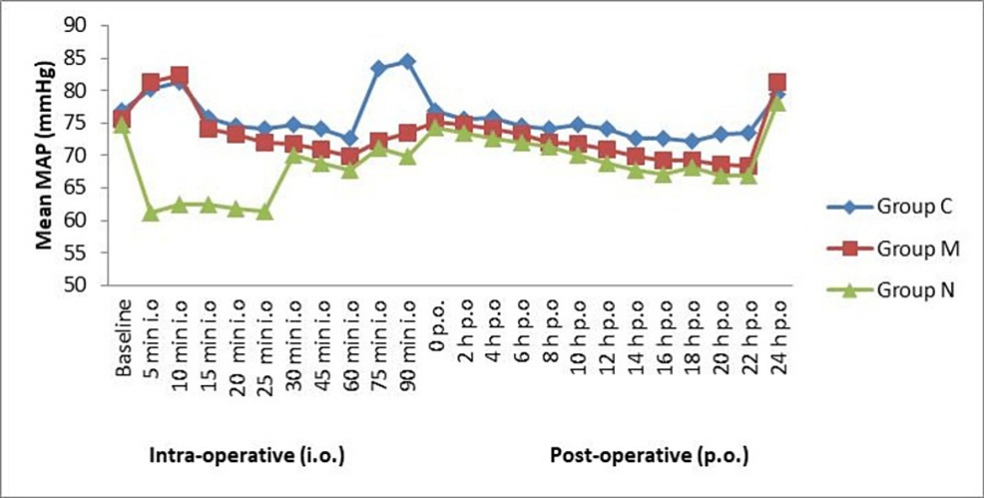
Mean MAP rate in intraoperative and postoperative periods (N = 90) MAP: mean arterial pressure.

The MAP was similar at 0 minutes and statistically significant (p > 0.05) among all the groups. The MAP was almost stable in the intraoperative period and postoperative period. No significant difference (p > 0.05) was observed among the groups in MAP at different time intervals (Figure 2).

**Figure 3:**
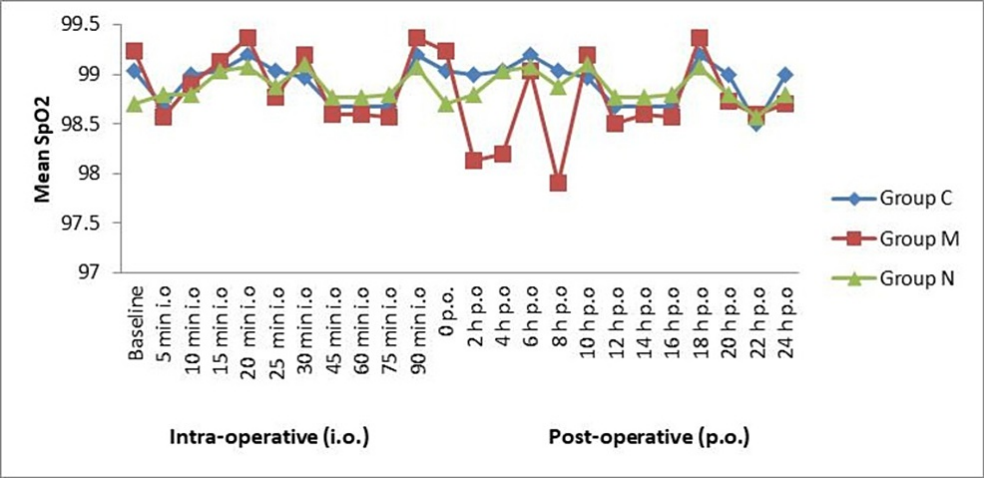
Mean SpO2 in intraoperative and postoperative periods (N = 90) SpO_2_: oxygen saturation.

The SpO_2_ was similar at 0 minutes among all the groups (p > 0.05). The SpO_2_ remained 97-100% in the intraoperative period and postoperative period among all the groups. No significant (p > 0.05) difference was observed among the groups in SpO_2_ at different time intervals (Figure 3).

The VAS was similar at zero hours, two hours, and four hours in the postoperative period among all the groups. There was a significant difference (p = 0.003) in VAS from six hours to 20 hours in the postoperative period among the groups. A significant difference (p < 0.05) was observed among the groups at eight hours to 20 hours (Table 2).

**Table 2:** Comparison of VAS score among the groups (N = 90) ^ 1 ^ANOVA test; * Significant; NA: not applicable; SD: standard deviation; VAS: visual analog scale.

Time period (hours)	Group C (N = 30)	Group M (N = 30)	Group N (N = 30)	p-value^1^
	Mean ± SD	Mean ± SD	Mean ± SD	
0	1.00 ± 0.00	1.00 ± 0.00	1.00 ± 0.00	NA
2	1.00 ± 0.00	1.00 ± 0.00	1.00 ± 0.00	NA
4	1.00 ± 0.00	1.00 ± 0.00	1.00 ± 0.00	NA
6	4.27 ± 0.45	1.00 ± 0.45	1.00 ± 0.37	0.003^*^
8	4.43 ± 0.63	2.43 ± 2.43	1.00 ± 1.23	0.002^*^
10	1.99 ± 1.49	3.70 ± 3.79	1.00 ± 1.02	<0.001^*^
12	1.29 ± 0.53	4.27 ± 0.99	0.99 ± 0.72	0.003^*^
14	4.45 ± 0.53	1.43 ± 1.99	4.30 ± 0.54	0.001^*^
16	4.53 ± 0.73	1.00 ± 1.94	4.33 ± 0.71	0.001^*^
18	1.00 ± 0.68	4.40 ± 3.99	1.63 ± 0.93	<0.001^*^
20	1.00 ± 0.21	1.43 ± 1.01	2.80 ± 0.41	0.02^*^
22	1.00 ± 0.33	1.33 ± 1.89	1.80 ± 1.06	0.08
24	1.00 ± 0.54	1.00 ± 1.01	1.00 ± 0.79	0.77

The RSS was similar at all the time intervals among all the groups in the postoperative period (Table 3).

**Table 3:** Comparison of RSS score among the groups (N = 90) ^1^ ANOVA test; * Significant; NA: not applicable; RSS: Ramsay Sedation Scale.

Time period (hour)	Group C (N = 30)	Group M (N = 30)	Group N (N = 30)	p-value^1^
	Mean ± SD	Mean± SD	Mean ± SD	
0	1.00 ± 0.82	1.77 ± 0.81	1.93 ± 0.45	0.09
2	1.00 ± 0.58	1.67 ± 0.35	3.53 ± 0.73	0.02*
4	1.00 ± 1.03	1.53 ± 0.45	2.30 ± 0.60	NA
6	1.00 ± 0.83	1.47 ± 0.68	1.97 ± 0.85	0.11
8	1.00 ± 0.96	1.30 ± 0.59	1.87 ± 1.14	0.10
10	1.00 ± 0.62	1.00 ± 0.26	1.80 ± 1.30	0.12
12	1.00 ± 0.46	1.00 ± 0.35	1.10 ± 1.03	0.14
14	1.00 ± 0.00	1.00 ± 0.37	1.03 ± 0.97	NA
16	1.00 ± 0.00	1.00 ± 0.64	1.00 ± 1.17	NA
18	1.00 ± 0.00	1.00 ± 0.37	1.80 ± 1.10	NA
20	1.00 ± 0.00	1.00 ± 0.37	1.10 ± 0.45	NA
22	1.00 ± 0.48	1.00 ± 0.25	1.00 ± 0.43	0.12
24	1.00 ± 0.48	1.00 ± 0.18	1.00 ± 0.45	0.13

A significant difference was found in the first requirement of rescue analgesia in the postoperative period among the groups (p < 0.001). The first requirement of analgesia was statistically significant (p = 0.001) and higher in Group N (7.70 ± 1.74) than in Group C (4.43 ± 1.43) and Group M (7.33 ± 2.21) (Table 4). A significant difference was found in the 24-hour requirement of rescue analgesia in the postoperative period among the groups (p = 0.001). The 24-hour consumption of rescue analgesia in the postoperative period was significantly (p = 0.001) lower in Group N (1.43 ± 0.50) than Group C (2.07 ± 0.64) and Group M (1.63 ± 0.55) (Table 4). Total consumption of antiemetics was among 16.3% of the patients in Group M only (Table 4).

**Table 4:** Comparison of the mean time of the first requirement of rescue analgesia and mean 24-hour consumption of rescue analgesia (paracetamol) in groups (N = 90) ^1 ^ANOVA test; * Significant, p = 0.001 (post hoc comparison test).

	Group C (N = 30)	Group M (N = 30)	Group N (N = 30)	p-value^1^
	Mean ± SD	Mean ± SD	Mean ± SD	
Time of the first requirement of rescue analgesia	4.43 ± 1.43	7.33 ± 2.21	7.70 ± 1.74	<0.001^*^
24-hour consumption of rescue analgesia (paracetamol)	2.07 ± 0.64	1.63 ± 0.55	1.43 ± 0.50	0.001^*^
	N (%)	N (%)	N (%)	
Consumption of antiemetic (ondansetron)	0 (0.0)	4 (16.3)	0 (0)	0.015^*^

Table 5 shows the comparison of side effects among all three groups. Hypotension was found among 6.6% in Group N only and respiratory depression in 6.6% of Group M patients. The itching was the same in Group M and Group N, i.e., 3.3%.

**Table 5:** Comparison of complications among the groups (N = 90) ^1 ^Chi-square test; NA: not applicable.

	Group C (N = 30)	Group M (N = 30)	Group N (N = 30)		p-value^1^
	N (%)	N (%)	N (%)		
Hypotension	0 (0.0)	0 (0.0)	2 (6.6)	0.129	
Respiratory depression	0 (0.0)	2 (6.6)	0 (0.0)	0.129	
Urinary retention	0 (0.0)	1 (3.3)	0 (0.0)	0.364	
Itching	0 (0.0)	1 (3.3)	1 (3.3)	0.600	
Shivering	0 (0.0)	0 (0.0)	0 (0.0)	NA	
Others	1 (3.3)	0 (0.0)	0 (0.0)	0.364	

## Discussion

Different types of surgical treatment are available for patients with breast cancer. Standard surgical procedures include lumpectomy, segmental mastectomy, total mastectomy, modified radical mastectomy, and radical mastectomy. GA is currently the standard technique used for the surgical treatment of breast cancer. However, the side effects and complications of GA preclude ambulatory surgery for most patients undergoing breast surgery. Nausea and vomiting after CA breast surgery with GA prolong recovery room stays and necessitate hospitalization for patients otherwise able to undergo ambulatory surgery [15]. Parenteral narcotic use is routine after emergence from anesthesia and during the early postoperative interval, which further increases the incidence of nausea, vomiting, and sedation and results in a prolonged recovery room and hospital stay.

Regional anesthesia using PVB is an ideal adjunct to GA for breast cancer surgery. The mechanism of action of paravertebral analgesia is the direct penetration of local anesthetic into the intercostal nerve, including its dorsal ramus, the rami communicating, and the sympathetic chain. The benefits of PVB include a reduction in postoperative nausea and vomiting, prolonged postoperative pain relief, and the potential for early discharge [16].

In this study, we observed that all three groups were comparable, and there was no statistically significant difference (p > 0.001) between the groups with respect to demographic characteristics of age, weight, height, body mass index, and ASA grading. The heart rate was similar at 0 minutes among the entire group (p > 0.05). The heart rate and MAP were almost stable with the time intervals. No significant (p > 0.05) difference was observed among the groups in heart rate at different time intervals, but hypotension occurred in two cases of clonidine, which was insignificant.

We assessed the VAS score in the postoperative period in all patients and observed that VAS was similar at zero hours, two hours, and four hours in the postoperative period among all the groups. However, a significant (p < 0.05) difference was observed among the groups from six hours to 20 hours. Group M had lesser VAS scores than Groups C and N, and the lowest VAS score was in Group M. This finding was in consonance with the other studies done so far in this field. Similarly, Klein et al. (2004) also observed that patients receiving PVB experienced statistically significantly less pain in comparison to patients receiving GA only [17]. Terheggen et al. (2002) observed that VAS scores in the postoperative period were significantly lower in patients who received PVB [18]. The maximum VAS score in the PVB group was 1115 mm versus 4423 mm in the GA group (p < 0.001). The PVB prior to GA results in lesser VAS scores in comparison to the control group [19]. Preoperative PVB seems to reduce the prevalence of chronic pain even after one year of breast cancer surgery. The patients who received PVB had significantly lower VAS than the group not receiving PVB [19].

The VAS score was used as the parameter for determining the requirement for rescue analgesia in the postoperative period. Patients reporting VAS scores of 3 or more were provided rescue analgesia with paracetamol intravenously. All the groups were compared for doses of paracetamol consumption. Group M was found to have a significantly lower consumption of paracetamol in grams (mean: 1.43 + 0.50) during the postoperative period than other groups, with the least requirement for rescue analgesia in Group M. In a study by Coveney et al. (1998), only 14 out of 112 patients receiving PVB (12.5%) required postoperative analgesia as compared to 72 out of the 89 (80.9%) patients who received GA (p < 0.0001).

Nausea and vomiting were observed in 20% and 50% of all operative procedures, respectively, and more so in female patients undergoing GA. The PONV in the PVB group was significantly lower (p = 0.026) in comparison to the placebo group [19]. The PONV in the PVB group at 24 hours postoperatively was significantly lower (p = 0.04) in comparison to patients receiving GA only [17]. Moller et al. (2007) observed that out of the 38 patients in the PVB group, only seven patients complained of PONV [20]. In their study of 60 patients, Sinha et al. (2012) found that group II (0.25% 18 ml ropivacaine with dexmedetomidine) significantly extended the duration of analgesia in PVB, and VAS were similar in the first few hours after surgery, but after that, VAS was much higher in group I [21]. The patients receiving PVB had a comparatively lesser incidence of PONV in comparison to the patients in the GA group [22]. In this study, a significant difference was found in the time of the first requirement for rescue analgesia in the postoperative period among the groups (p = 0.001). The time of the first requirement for rescue analgesia in the postoperative period was significantly (p = 0.001) higher in Group N (7.70 + 1.74) than in Group M (7.33 + 2.21). Fentanyl and clonidine in combination with low-dose ropivacaine have superior analgesic efficacy to plain ropivacaine [11]. In this study, a significant difference was found in the level of sedation among the groups. The sedation score is higher in Group C. Similarly, Huang et al. (2007) observed that clonidine is associated with a significant increase in postoperative sedation within 24 hours [23]. The numeric rating scale scores of all groups were compared. Observations were recorded at two-hour intervals until 24 hours postoperatively. Similarly, another study involving 25 patients observed that out of the 17 patients receiving PVB, 13 patients had no nausea or vomiting in the entire postoperative period [24]. Similarly, Sharma et al. (2013) observed no statistical significance in PONV with multilevel PVB with bupivacaine 0.5% and ropivacaine [25].

Various studies on PVBs have quoted different rates of complications. No complications were reported by Greengrass et al. [24]. Coveney et al. (1998) reported complications in 2.6% of patients, with two cases experiencing epidural extension and one patient developing pneumothorax [22,24]. Terheggen et al. (2002) reported one case of epidural block and one patient with a pleural puncture [18]. Sharma et al. (2013) reported that none of the patients had any complications in the first 24 hours of the postoperative period [25].

In the present study, no complications related to the procedure technique were noted in any of the groups. There was no evidence of pneumothorax, hematoma, total spinal anesthesia, or local anesthetic toxicity. Hence, PVB can be considered a safe adjunct to GA. In the present study, Group N had better patient satisfaction and early recovery than Groups M and C. Our study is similar to those studies where the use of preoperative PVB in patients undergoing mastectomy plus immediate reconstruction significantly decreased patient length of stay [26]. Ropivacaine 0.5% provides a good patient satisfaction score after a multilevel thoracic PVB [25].

Limitations

The unavailability of different age groups was present in this study. Due to this, the precision of this result on different age groups could not be figured out. We found that patients who received morphine for PVB were more comfortable and had a longer duration of analgesia but this was not statistically significant.

## Conclusions

This study was conducted to know the efficacy, safety, and postoperative analgesic effects of morphine and clonidine as adjuncts to ropivacaine PVB during modified radical mastectomy. We concluded that morphine provides superior analgesia in the postoperative period than clonidine and ropivacaine. PVB reduces the incidence of postoperative nausea and vomiting and this was statistically significant in morphine. In the morphine group, there was a significantly reduced consumption of rescue analgesia in the postoperative period in comparison to clonidine and ropivacaine. In the clonidine group, the pain was significantly delayed in the postoperative period as compared to morphine and ropivacaine. The ropivacaine group had better hemodynamic stability than morphine and clonidine. The clonidine group had better patient satisfaction than morphine and ropivacaine.
